# Effectiveness of message framing on women’s intention to perform cytomegalovirus prevention behaviors: a cross-sectional study

**DOI:** 10.1186/s12905-017-0492-x

**Published:** 2017-12-20

**Authors:** Rosemary Thackeray, Brianna M. Magnusson, Emily M. Christensen

**Affiliations:** Department of Health Science, Brigham Young University, 4103 LSB, Provo, UT 84602 USA

**Keywords:** Cytomegalovirus, Message framing, Health communication, Infection, Pregnancy, Intention

## Abstract

**Background:**

The purpose of this study was to evaluate the effect of message framing on women’s intention to perform cytomegalovirus (CMV) prevention behaviors involving handwashing, not sharing food and eating utensils, not kissing a child on the lips and not placing a pacifier in the mouth after it was in a child’s mouth.

**Methods:**

An online panel of women 18–40 years, who were pregnant or planning a pregnancy were randomized in a 2 × 2 factorial design to receive 1 of 4 CMV fact sheets. The fact sheets were framed as either what could be gained or be lost by following (or not) the recommendations and the likelihood of being affected by CMV (i.e., small chance or one of the most common infections in infants). The questionnaire measured CMV knowledge, participation in CMV risk or prevention behaviors, perceived severity of and susceptibly to CMV, and the perceived control over and the efficacy of recommended prevention behaviors. The dependent variable, intention to modify behavior, was an index score that ranged from 0 to 16 with higher values indicating greater intention. Linear regression was used to evaluate the association between all independent variables and overall behavioral intention.

**Results:**

The sample included 840 women; 15.5% were familiar with CMV. Behavioral intention was high (*M* = 10.43; *SD* = 5.13) but did not differ across the message frames (*p* = 0.23). Overall, behavioral intention was predicted by CMV knowledge, message credibility, perceived severity of CMV, perceived behavioral control and response efficacy. Significant interactions with gain vs. loss frame were observed for perceived behavioral control (*p* = 0.03) and response efficacy (*p* = .003).

**Conclusions:**

Framing CMV messages by what women stand to gain or lose interacts with perceived behavioral control and response efficacy to influence behavioral intention. Perceived behavioral control and response efficacy were most predictive of behavioral intention overall regardless of frame. Messaging that focuses on these two variables, particularly for avoiding kissing a child on the lips and sharing food, cups and utensils, may result in greater gains in intention to participate in CMV prevention behaviors.

## Background

In the United States, congenital cytomegalovirus (CMV) infection is one of the most common causes of birth defects and developmental delays in infants. It is the leading cause of infant hearing loss [1] and can also result in cognitive and motor deficits, vision loss, or death [2]. Each year, there are nearly 26,000 U.S. children born with congenital CMV for a total birth prevalence of 0.64% [3]. Of these, approximately 400 infants will die and 8000 will develop lifelong disabilities [4]. Although the effects of this condition are serious, awareness of CMV among adults remains low at 13–39% [5–7].

Infants born with CMV are most commonly infected with the virus through transmission from mother to fetus during pregnancy [8]. The virus can also be transmitted through sexual contact, breastmilk, and organ transplantation [9]. Young children are the primary carriers of the virus [3]. If a woman is exposed to CMV for the first time prior to conception or at any time during pregnancy her infant may become infected [9]. Because no effective, licensed, CMV vaccine exists [10], behavioral practices are the only means for women to limit their exposure to CMV or reduce CMV transmission. Handwashing is currently the primary behavioral recommendation for reducing CMV transmission [11, 12]. However, research has shown that behaviors that limit a woman’s contact with children’s saliva may be especially efficacious in reducing infection [13]. These include avoiding contact with saliva when kissing a child and not putting things in one’s mouth that have been in a child’s mouth (specifically food, cups, forks or spoons, or pacifiers) [14]. Past research has shown that women regularly participate in behaviors that increase their risk of CMV infection through direct saliva sharing [15, 16]. Women also regularly engage in the protective behavior of handwashing [15, 16].

There is relatively limited research on how to effectively communicate CMV prevention information. Individual-level interventions, specifically healthcare provider counseling, have been shown to be effective in reducing CMV infection [17–19]. One study found that a fact sheet and video both encouraged women to practice individual prevention behaviors, look for more information about CMV, and share their knowledge with family members or friends [16]. There is no published research on the type of CMV message content that is most effective. Therefore, the purpose of this research was to explore the influence of message framing on women’s intentions to perform CMV prevention behaviors.

### Message framing

Message framing refers to a common health communication technique in which behavior change messages are focused on the potential outcomes of a proposed health behavior. The theoretical basis for message framing stems from behavioral economic prospect theory [20]. Prospect theory’s premise is that when presenting a person with a choice between two alternatives, framing the choice in terms of the potential losses (cost of not complying) or gains (benefits) has differential effects on decision making and subsequent behavior. Gain and loss framed messages are structured around two components: 1) the outcomes or consequences of taking action or not taking action, and 2) the possibility that the specified outcome will be obtained, or not obtained [21]. Gain-framed messages focus on obtaining a desirable outcome (“If you quit smoking your lungs will be healthy and strong,”) or avoiding an undesirable outcome (“If you quit smoking you will be less likely to get lung cancer.”). Loss-framed messages emphasize being the recipient of the undesirable outcome (“If you do not quit smoking you may develop gum disease and lose your teeth,”) or failing to receive a desirable outcome by not following the advocated behavior (“If you do not quit smoking, you may not be able to keep your original teeth.”)

Message framing research suggests that the influence of gain and loss messages on advocated behaviors can be direct. In other words, the person can change behavior after hearing or seeing a message. Or it can be mediated by additional variables, one of which is behavioral intention [22]. Rogers [23] suggested that intention to perform a behavior results from an individual’s appraisal of three factors: severity of the event or outcome; perceived probability of the event occurring (susceptibility, or level of risk), and belief of whether following the recommended behavior will yield the desired result (response efficacy).

Additionally, research indicates that when a person perceives that individual risk is high, a loss frame tends to be more effective at influencing behavioral intention [24]; when risk perception is low people respond more positively to gain framed messages aimed at influencing behavioral intention [25] and actual behavior [26]. In some cases, among those who perceive they are not at risk, framing makes no difference in behavioral intention [24].

For response efficacy, studies have shown that loss frame is more effective at influencing behavioral intention for behaviors with low response efficacy [27, 28]. When there is high response efficacy, framing either has no effect on intention [27, 28] or gain frame messages are more persuasive [29–31]. Similarly, with high response efficacy, gain framed messages are more persuasive for actual behavior change [29].

In addition, the function of the proposed behavior can moderate the effect of message framing on intention and behavior [22]. Health behaviors are classified as having one of two functions, also known as roles: detecting potential health problems (e.g. mammography for breast cancer detection) or preventing future health issues (e.g. vaccination), also called protection behaviors [21]. Research on the interaction of behavioral function and message framing suggests that gain-framed messages may be more persuasive for protection-related behaviors [32, 33].

Given that CMV awareness is low, that there are significant consequences of CMV infection in infants, and that there is a scarcity of research in how to communicate this information, this study aimed to look at the effect of framed CMV messaging among women of childbearing years. Specifically we were interested in the association between message framing and behavioral intention for CMV prevention behaviors.

There were three hypotheses.

Hypothesis 1: Because the messages are focused on prevention-related behaviors, overall, gain-framed messages will result in greater intention to engage in CMV prevention behaviors.

Hypothesis 2: Messages that frame the potential risk or susceptibility of CMV infection as the most common infection will be associated with greater intention to engage in CMV prevention behaviors.

Hypothesis 3: For the group of women who perceive they are at risk for CMV infection, the loss frame will result in greater intention to engage in CMV prevention behaviors.

## Method

### Procedure and stimuli

This was a cross-sectional descriptive study using a 2 × 2 factorial design. Two likelihood conditions were chosen because the odds of being affected by CMV are low, yet CMV infection is one of the most common infections that can affect a baby [2]. A single outcome was framed as either what they would gain by practicing or lose by failing to practice the CMV prevention behaviors. This was chosen based on the message framing literature. The stimuli were four, one-page fact sheets about CMV which included research-supported behavioral recommendations from a previous study [16]. The text describing CMV and how it is spread was identical within each frame. Table 1 shows the key message content for each of the four frames.

**Table 1:** Critical content of the CMV fact sheets: Four framed messages

Frame	Gain Frame		Loss Frame	
	Small Chance	Most Common	Small Chance	Most Common
Likelihood of infection	Small chance that you will get infected. Of every 1000 babies born only 6 will get a CMV infection from his/her mother.	One of the most common infections in babies. 1 in 150 babies is born with a CMV infection.	Small chance that you will get infected. Of every 1000 babies born only 6 will get a CMV infection from his/her mother.	One of the most common infections in babies. 1 in 150 babies is born with a CMV infection.
Behaviors that increase or decrease chances	Behaviors that decrease your chances of CMV infection:		Behaviors that increase your chances of CMV infection:	
	Do not kiss a young child on the lips. Do not share food, cups and eating utensils with a young child. Do not put a pacifier in your mouth after it has been in your child’s mouth. Wash your hands after changing a diaper or wiping a nose.		Kiss a young child on the lips. Share food, cups and eating utensils with a young child. Put a pacifier in your mouth after it has been in your child’s mouth. Forget to wash your hands after changing a diaper or wiping a nose	
Benefits/Costs	Benefits you will gain by following these behavior recommendations:		Costs you will pay by doing these behaviors:	
	You decrease your chances of getting a CMV infection. If you do not get CMV while pregnant you will not pass CMV to your unborn baby. You will decrease the chance of having a baby born with severe birth defects.		You increase your chances of getting a CMV infection. If you do get CMV while pregnant you can pass CMV to your unborn baby. You will increase the chance of having a baby born with severe birth defects.	

The fact sheets were pretested with a convenience sample of 113 women during four rounds of testing. Revisions were made to the fact sheets and messages for clarity and comprehension. The stimulus was embedded in a web-based survey and each respondent was randomized to receive one of the four fact sheets.

### Sample

The sample included women aged 18–40 years who had a child 5 years of age or younger at home and were currently pregnant or planning to become pregnant within 12 months. The national United States sample was recruited during 2015 from an online panel managed by Qualtrics, a worldwide software research company. Women who had ever worked as a healthcare provider and those who had a child with a previously diagnosed disability were excluded as both groups were likely to have higher CMV awareness than the general population. Respondents were compensated in reward points credited to their Qualtrics account. The Brigham Young University institutional review board approved the study.

A sample size of 800 was selected so that we were able to have 200 in each cell (2 × 2 study design). The sample sizes in the message framing literature vary and average around 100 participants [27]. For our study, to estimate the proportion of women who know about CMV we needed a sample size of 384.

Two quality control measures were used during data collection. The first measure was a minimum time frame the respondent spent viewing the message. The second asked respondents to type in a specific word. Respondents who failed to meet the quality control criteria were excluded from the sample and were replaced until quotas were met.

Framing manipulation checks included four questions. These questions assessed whether the respondent had read and understood the fact sheet. The questions also measured whether survey respondents differentially perceived the messages in the four versions of the fact sheet.

### Instrumentation

#### Outcome variable

The overall outcome variable was intention to perform the CMV prevention or risk behaviors. Behavioral intention was measured by one item for each of the eight behaviors. Respondents were asked how often they would engage in the behavior after reading the CMV fact sheet as compared to what they did before [15]. Responses were on a five point Likert scale from a lot less often to a lot more often. The optimal desired direction (either less or more) varied because to reduce risk, some behaviors should be less frequent (i.e. sharing food and utensils,) while others such as handwashing should be performed more often.

#### Predictor variables and constructs

The questionnaire assessed predictor variables selected based on past research with message framing studies: demographics, CMV awareness, message persuasiveness, and frequency of practicing CMV risk behaviors. Six constructs were also measured as detailed in Table 2 and in the following paragraphs. For each of the constructs an index score was created. For all scales, higher values indicated more of the construct. Demographic characteristics included education, household income, race/ethnicity, age of the youngest child at home, and pregnancy status.

**Table 2:** Psychometric Properties and Descriptive Statistics for Constructs Overall and by Message Frame

	Total Sample	Small Chance Gain Frame	Most Common Loss Frame	Small Chance Loss Frame	Most Common Gain Frame	*p*-value	Number of Items in the Scale	Possible Range	Cronbach’s Alpha
	*N* = 840	*N* = 211	*N* = 211	*N* = 211	*N* = 207				
	*M* (*SD*)								
Knowledge Scale^a^	3.28 (2.66)	3.44 (2.77)	3.24 (2.66)	3.13 (2.51)	3.32 (2.69)	0.689	12	0–12	0.82
Message Credibility^b^	5.57 (1.11)	5.58 (1.15)	5.47 (1.15)	5.51 (1.14)	5.73 (0.99)	0.087	3	1–7	0.89
Perceived Severity^b^	6.09 (1.04)	6.02 (1.10)	6.04 (0.99)	6.09 (1.08)	6.20 (0.96)	0.294	3	1–7	0.90
Perceived Susceptibility^b^	4.07 (1.36)	3.93 (1.31)	4.16 (1.37)	4.16 (1.28)	4.03 (1.45)	0.243	3	1–7	0.81
Response Efficacy^c^	4.46 (0.65)	4.43 (0.72)	4.43 (0.62)	4.51 (0.62)	4.49 (0.64)	0.482	8	1–5	0.93
Perceived Behavioral Control^b^	6.04 (0.94)	6.00 (0.95)	5.93 (0.95)	6.06 (0.93)	6.16 (0.92)	0.083	16	1–7	0.93

^a^Higher values indicate higher levels of CMV knowledge

^b^Responses on a 7-point Likert scale where 1 = Strongly Disagree and 7 = Strongly Agree

^c^Responses on a 5-point Likert scale where 1 = Not at all effective and 5 = Very effective

CMV awareness was assessed by asking respondents to rate their level of familiarity with CMV [16] and whether their healthcare provider had ever talked to them about CMV (yes or no). CMV background knowledge was measured by 12 items about CMV [16]. Response options were true (*n* = 6), false (*n* = 5) and I don’t know. All questions were coded 1 if correct and 0 if incorrect or don’t know. For each of the eight CMV prevention behaviors respondents were asked to rate on a 5-point Likert scale how often they performed or participated in each behavior [16]. Anchors for the three handwashing questions were never/always, while the scales for the remaining behaviors were ranged from never to every day.

Persuasiveness of the fact sheet material was assessed by asking respondents to indicate their agreement (strongly agree to strongly disagree) with one item, “If I were pregnant I would try to avoid catching CMV as a result of viewing this fact sheet” [16]. Message credibility was measured with three questions adapted from Regan et al. [34] which asked respondents to indicate their agreement that the fact sheet information was accurate, believable, and credible.

Perceived severity of CMV infection was measured with three questions adapted from Block and Keller [27] about the degree to which the respondent felt CMV infection in a baby was frightening, dangerous, or severe. Perceived susceptibility of CMV infection was measured by three items adapted from Nan [28] in which respondents indicated their level of agreement regarding the perceived likelihood, possibility of infection, and risk of getting CMV. Respondents answered one additional question about their perceived level of risk [35].

Perceived response efficacy of each of the CMV prevention behaviors was measured by one item adapted from Taber and Aspinwall [36]. The question asked how effective the respondent thought each behavior would be at decreasing their risk of getting CMV. CMV prevention behaviors, specifically those related to sharing with a child, inherently require the child’s cooperation. As such, respondents were asked to evaluate the extent to which they felt confident they could modify these behaviors (perceived behavioral control) and that it would be possible to do so [35].

### Statistical analysis

#### Descriptive analysis

Frequencies, proportions, and scale means and spread were calculated to describe the sample’s sociodemographic characteristics, background knowledge, frequency of practicing CMV prevention or risk behaviors and the remaining constructs displayed in Table 2. The chi-square test was used to test for the difference in proportions across the frames and ANOVA was used to compare means across the frames. The framing condition was operationalized into two variables: 1) gain or loss frame and 2) small chance/most common.

#### Total score for overall behavioral intention

To measure the outcome variable, behavioral intention, the responses were re-coded and categorized as a three level variable with values of 0 (respondent behavior would remain the same), 1 (change behavior “slightly more/less” in the desired direction), and 2 (change behavior “a lot more/less” in the desired direction). Respondents who stated that they would change their behavior in the opposite of the desired direction (e.g., wash hands less; *n* = 23–43) were also classified as 0, no change in behavior. The 3-level items were then summed to create a behavioral intention index score ranging from 0 to 16 where higher values indicated greater intention. The resulting index score had a Cronbach’s alpha of 0.92.

#### Linear regression analysis- overall behavioral intention

Linear regression was used to evaluate the relationship of the independent variables on the overall behavioral intention scale. All variables were entered into the model as main effects and, as we were interested in testing potential moderating effect of framing conditions, all constructs were entered into the model as a two-way interaction with each of the two framing variables. To create a parsimonious model that adequately controlled for confounding, backward elimination was used to individually remove variables and interaction effects that did not achieve significance at the α = 0.10 level. Consistent with principles of hierarchically well-formulated models, non-significant main effects were retained if they were part of a significant interaction term.

#### Logistic regression analysis – Intention to change individual behaviors

To determine if the associations observed in the linear regression model varied across the CMV prevention behaviors, the 3-level behavioral intention index scores, were used as the dependent variable for multinomial logistic regression. The constructs and interactions that were significant in the linear regression model were included. Additionally, based on an a priori hypothesis that frequency of participation in behavior would affect behavioral intention, pre-survey frequency of performing the behavior was also included. All analyses were conducted in SAS 9.4 (SAS Institute, Inc., Cary, NC, USA).

## Results

There were 848 respondents. Of these, eight women incorrectly indicated that CMV could be caught from a mosquito and they were eliminated from further analysis for a final sample of 840. The mean age of women in the sample was 28.8 years (SD: 4.64). The majority of respondents (72.7%) were White, non-Hispanic and 40.4% reported having a college degree while 21.1% had a high school education or less. The plurality of women (32.5%) reported incomes in the range of $25,000 to less than $50,000. A quarter (25.6%) of women in the sample had income greater than $75,000 and 14% reported income <$25,000. Nearly half (47.5%) of the sample was currently pregnant and most women (67.0%) had a child 2 years or younger. Only 15.5% of women reported any familiarity with CMV and 6.1% had talked to a healthcare provider about CMV. There were no significant differences between the message frames with regard to demographics or CMV background knowledge.

Results indicate that the message framing conditions were manipulated effectively. There were statistically significant differences between the gain and loss message framing for both tone of the fact sheet and whether the fact sheet stressed the benefits of following the recommended behaviors (*p* < .001). The majority of respondents in each message frame correctly identified that there was a small chance of getting CMV (*n* = 326; 77.2%) or if CMV was one of the most common infections in newborn babies (*n* = 309; 73.9%).

**Fig. 1:**
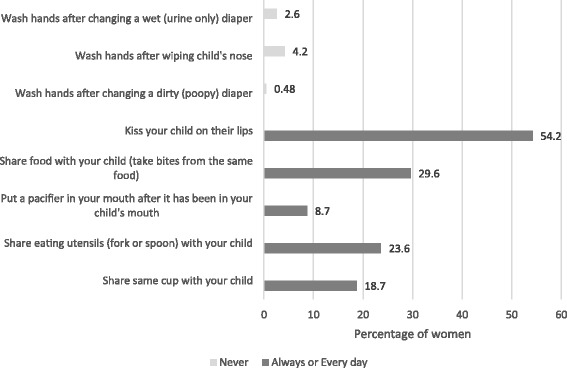
Percentage of respondents participating in CMV risk and prevention behaviors prior to the survey

The distribution of women in the highest risk behavioral groups for pre-survey participation in CMV risk and prevention behaviors is displayed in Fig. 1. CMV risk behaviors were common in the population with the exception of putting a pacifier in one’s mouth after it had been in a child’s mouth, which was reported at least weekly by only 25.71% of the sample. There were no differences across the four frames in the mean score for frequency of practicing each behavior (data not shown).

**Fig. 2:**
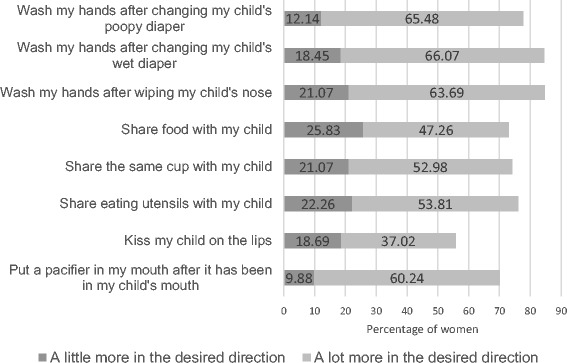
Respondents reporting that they intended to change their behavior after viewing the CMV fact sheet. To prevent CMV, handwashing behaviors should increase, while all other behaviors should decrease

The percentage of women who reported they would change each behavior is displayed in Fig. 2. Regardless of behavior, the majority of women indicated they would change their behavior after viewing the fact sheet. However, intention for kissing was lower as only 55.7% of women reported intention to decrease the frequency of kissing their child on the lips. The behavioral intention index score across all eight behaviors had a mean value of 10.43 (*SD* = 5.13).

### Message credibility and persuasiveness

The mean score for message credibility was not significantly different across the four message frames (Table 2). However, when examining the gain or loss framed messages separately, regardless if the message included the small chance or most common message, credibility scores were significantly different between gain frame (*M* = 5.65, *SD* = 1.08) and loss frame (*M* = 5.50, *SD* = 1.14; *p* = .03).

Women in the sample overwhelmingly stated they found the fact sheets persuasive. Nearly all respondents (93.9%; *n* = 789) reported they would try to avoid contracting CMV as a result of viewing the fact sheet. There were no statistically significant differences in persuasiveness across the frames (*p* = 0.32; data not shown).

### Effects of message framing on behavioral intention

It was hypothesized that both the gain frame (hypothesis 1) and the most common frame (hypothesis 2) would be associated with increases in behavioral intention. However, no main effects on behavioral intention were observed with either the gain vs. loss or the small chance vs. most common message framing conditions. Two constructs had significant interactions with the gain vs. loss frame, specifically response efficacy and perceived behavioral control. No interaction effects with the small chance vs. most common frame achieved significance in the model. Additionally, perceived susceptibility and perceived level of risk failed to achieve significance in the model. The final regression model was adjusted for gain frame vs. loss frame, CMV knowledge, message credibility, perceived severity, perceived behavioral control and response efficacy along with the two interaction terms. The results of the linear regression are presented in Table 3. Overall, response efficacy and perceived behavioral control were most strongly associated with behavioral intention and appear to interact with gain and loss message framing.

**Table 3:** Multiple linear regression identifying factors associated with increased intention to modify CMV risk and prevention behaviors in the desired direction

	*n* = 840	
Model r2	0.39	
Variable	b (SE)	*p*-value
Intercept	−15.73 (1.57)	<0.001
Main Effects		
Gain Frame	2.22 (2.07)	0.28
Knowledge Scale^a^	0.15 (0.05)	0.006
Message Credibility^b^	0.29 (0.15)	0.06
Perceived Severity^b^	0.56 (0.15)	<0.001
Response Efficacy^c^	3.19 (0.44)	<0.001
Perceived Behavioral Control^b^	1.05 (0.29)	0.000
Interaction Effects		
Response Efficacy x Gain Frame	−1.69 (0.58)	0.003
Perceived Behavioral Control x Gain Frame	0.90 (0.40)	0.03

Note: Behavioral Intention was measured using a score ranging from 0 to 16. The score was created by summing intention scores for each behavior where those intention scores equaled 0 if the respondent intended to “remain the same”; 1 if the respondent intended to change their behavior “a little” in the desired direction; and 2 if the respondent intended to change their behavior “a lot” in the desired direction. To prevent CMV, handwashing behaviors should increase, while all other behaviors should decrease

^a^Higher values indicate higher levels of CMV knowledge

^b^Responses on a 7-point Likert scale where 1 = Strongly Disagree and 7 = Strongly Agree

^c^Responses on a 5-point Likert scale where 1 = Not at all effective and 5 = Very effective

### Relationship between message frame and predictor variables and constructs

There were no significant differences between the four message frames for any of the variables or constructs (see Table 2). Similarly, no significant differences were observed on any of the six constructs when comparing small chance vs. most common irrespective of whether it included gain or loss frames (data not shown). When comparing the six constructs across gain and loss frames, regardless of small chance or most common frame, perceived susceptibility is marginally significant (*p =* 0.06*)* with those in the loss frame (*M* = 4.16, *SD* = 1.33) reporting higher mean levels of susceptibility than those in the gain frame (*M* = 3.98, *SD* = 1.38).

### Predictors of behavioral intention

Perceived behavioral control and response efficacy were associated with the largest increases in overall behavioral intention (See Table 3). Given that significant interaction effects between gain and loss frame were observed for both behavioral control and response efficacy, these should be interpreted with consideration of framing. Regardless of frame, perceived behavioral control is associated with an increase in behavioral intention, however this effect is more pronounced among respondents in the gain frame. Conversely, the effect of response efficacy, although positively associated with behavioral intention in both frames, is more pronounced among those in the loss frame.

Recognizing that overall behavioral intention for reducing CMV risk requires a woman to engage in multiple behaviors and women may be more motivated to modify some than others, intention for each of the eight behaviors was examined individually. The logistic regression results for each of the eight individual CMV prevention behaviors are displayed in Tables 4, 5, and 6. Similar to the overall behavioral intention, perceived behavioral control and response efficacy were strongly associated with intention for individual behaviors. Although confidence intervals overlap for both handwashing and sharing behaviors, the association between perceived behavioral control and intention to change behavior “a lot” in the desired direction is stronger among those in the gain frame. Similarly, for both handwashing and sharing behaviors, the association between response efficacy and intention to change behavior “a lot” was stronger for the loss frame.

**Table 4:** Logistic regression predicting the odds of changing CMV handwashing prevention behavior in the desired direction

	Washing hands after changing a poopy diaper		Washing hands after changing a wet diaper		Washing hands after wiping a child’s nose	
	A Lot More Often	A Little More Often	A Lot More Often	A Little More Often	A Lot More Often	A Little More Often
	*n* = 550	*n* - 102	*n* = 555	*n* = 155	*n* = 535	*n* = 177
	OR (95% CI)		OR (95% CI)		OR (95% CI)	
Pre-Survey Behavior Frequency^a^	**1.79 (1.32–2.42)**	**3.17 (2.25–4.48)**	**1.36 (1.11–1.67)**	**1.65 (1.32–2.08)**	**1.26 (1.02–1.54)**	**1.54 (1.24–1.92)**
Knowledge Scale^b^	**1.14 (1.06–1.23)**	1.11 (0.99–1.23)	**1.12 (1.02–1.22)**	1.06 (0.95–1.17)	1.06 (0.97–1.16)	1.02 (0.92–1.12)
Message Credibility^c^	**1.22 (1.01–1.46)**	1.14 (0.88–1.47)	1.21 (0.99–1.49)	1.22 (0.95–1.56)	1.20 (0.96–1.50)	0.97 (0.77–1.23)
Perceived Severity^c^	**1.31 (1.08–1.59)**	0.88 (0.69–1.14)	1.24 (1.00–1.53)	1.01 (0.80–1.29)	**1.29 (1.03–1.62)**	1.01 (0.80–1.27)
Response Efficacy^d,e^ x Gain Frame	**1.95 (1.21–3.14)**	1.62 (0.87–3.00)	**2.12 (1.35–3.32)**	1.40 (0.88–2.23)	**2.19 (1.44–3.31)**	1.43 (0.96–2.12)
Response Efficacy^d,e^ x Loss Frame	**3.07 (1.88–5.00)**	1.09 (0.64–1.86)	**2.28 (1.50–3.47)**	1.37 (0.89–2.10)	**2.63 (1.70–4.08)**	1.07 (0.71–1.61)
Perceived Behavioral Control^c,e^ x Gain Frame	1.24 (0.76–2.02)	**0.48 (0.28–0.83)**	**1.76 (1.23–2.52)**	0.92 (0.65–1.31)	**2.22 (1.61–3.06)**	1.11 (0.82–1.49)
Perceived Behavioral Control^c,e^ x Loss Frame	1.24 (0.85–1.81)	1.23 (0.80–1.89)	**1.59 (1.14–2.21)**	0.87 (0.63–1.22)	**1.91 (1.38–2.63)**	1.30 (0.95–1.77)

Note. Confidence intervals that are statistically significant are bolded

^a^Participation in the behavior that is being modeled. Handwashing behaviors are reverse coded such that a one-step decrease in frequency of handwashing (e.g. from most of the time to some of the time) is associated with and increased odds of intention to change behavior

^b^Higher values indicate higher levels of CMV knowledge

^c^Responses on a 7-point Likert scale where 1 = Strongly Disagree and 7 = Strongly Agree

^d^Behavior-specific perceived behavioral control and response efficacy use for each model

^e^Responses on a 5-point Likert scale where 1 = Not at all effective and 5 = Very effective

**Table 5:** Logistic regression predicting the odds of changing CMV sharing prevention behaviors in the desired direction

	Sharing food with a child		Sharing cups with a child		Sharing utensils with a child	
	A Lot Less Often	A Little Less Often	A Lot Less Often	A Little Less Often	A Lot Less Often	A Little Less Often
	*n* = 397	*n* = 217	*n* = 445	*n* = 177	*n* = 452	*n* = 187
	OR (95% CI)		OR (95% CI)		OR (95% CI)	
Pre-Survey Behavior Frequency^a^	0.82 (0.70–0.98)	0.98 (0.83–1.16)	0.96 (0.83–1.11)	**1.19 (1.02–1.38)**	0.92 (0.79–1.07)	1.14 (0.96–1.34)
Knowledge Scale^b^	1.06 (0.97–1.15)	1.03 (0.95–1.12)	1.07 (0.99–1.15)	1.00 (0.91–1.08)	1.03 (0.95–1.11)	0.99 (0.90–1.07)
Message Credibility^c^	**1.31 (1.06–1.61)**	1.16 (0.94–1.42)	**1.27 (1.03–1.55)**	1.14 (0.92–1.41)	**1.43 (1.17–1.76)**	**1.28 (1.03–1.59)**
Perceived Severity^c^	**1.56 (1.25–1.94)**	1.19 (0.98–1.45)	**1.39 (1.13–1.70)**	**1.32 (1.06–1.63)**	**1.37 (1.12–1.69)**	**1.25 (1.01–1.55)**
Response Efficacy^d,e^ x Gain Frame	**2.49 (1.74–3.56)**	1.24 (0.94–1.64)	**2.69 (1.85–3.90)**	1.11 (0.80–1.54)	**2.42 (1.71–3.44)**	1.13 (0.83–1.53)
Response Efficacy^d,e^ x Loss Frame	**3.19 (2.19–4.63)**	**1.54 (1.15–2.05)**	**3.63 (2.37–5.56)**	1.24 (0.87–1.76)	**3.28 (2.18–4.94)**	**1.69 (1.19–2.41)**
Perceived Behavioral Control^c,e^ x Gain Frame	**2.23 (1.76–2.81)**	**1.32 (1.10–1.59)**	**1.91 (1.49–2.43)**	1.12 (0.90–1.39)	**1.91 (1.50–2.42)**	1.22 (0.98–1.52)
Perceived Behavioral Control^c,e^ x Loss Frame	**1.81 (1.44–2.28)**	1.06 (0.89–1.27)	1.57 (1.20–2.05)	1.06 (0.84–1.34)	**1.57 (1.22–2.03)**	0.94 (0.75–1.17)

Note. Confidence intervals that are statistically significant are bolded

^a^Participation in the behavior that is being modeled. The odds ratio shown is for a one-step increase in participation of behavior (e.g. from 1 to 2 days per week to 3–5 days per week)

^b^Higher values indicate higher levels of CMV knowledge

^c^Responses on a 7-point Likert scale where 1 = Strongly Disagree and 7 = Strongly Agree

^d^Behavior-specific perceived behavioral control and response efficacy use for each model

^e^Responses on a 5-point Likert scale where 1 = Not at all effective and 5 = Very effective

**Table 6:** Logistic regression predicting the odds of changing CMV kissing on the lips and pacifier use prevention behaviors in the desired direction

	Kissing a child on the lips		Putting a pacifier in your mouth	
	A Lot Less Often	A Little Less Often	A Lot Less Often	A Little Less Often
	*n* = 311	*n* = 157	*n* = 506	*n* = 83
	OR (95% CI)		OR (95% CI)	
Pre-Survey Behavior Frequency^a^	0.87 (0.76–1.00)	**1.26 (1.05–1.51)**	1.06 (0.93–1.22)	**1.62 (1.34–1.96)**
Knowledge Scale^b^	1.07 (1.00–1.15)	0.98 (0.91–1.06)	1.03 (0.97–1.10)	0.98 (0.87–1.09)
Message Credibility^c^	1.20 (0.98–1.46)	**1.36 (1.10–1.68)**	1.19 (1.00–1.41)	0.80 (0.62–1.04)
Perceived Severity^c^	**1.43 (1.16–1.77)**	1.12 (0.91–1.38)	**1.41 (1.18–1.68)**	1.09 (0.84–1.40)
Response Efficacy^d,e^ x Gain Frame	**1.68 (1.28–1.20)**	1.25 (1.00–1.58)	**2.08 (1.43–3.03)**	**1.85 (1.09–3.15)**
Response Efficacy^d,e^ x Loss Frame	**1.50 (1.13–2.00)**	1.16 (0.92–1.46)	**1.84 (1.28–2.64)**	1.44 (0.91–2.28)
Perceived Behavioral Control^c,e^ x Gain Frame	**1.69 (1.44–1.98)**	1.16 (1.00–1.34)	1.23 (0.93–1.63)	0.68 (0.48–0.97)
Perceived Behavioral Control^c,e^ x Loss Frame	**1.82 (1.53–2.16)**	1.27 (1.09–1.47)	**1.35 (1.03–1.78)**	0.83 (0.61–1.13)

Note. Confidence intervals that are statistically significant are bolded

^a^Participation in the behavior that is being modeled. The odds ratio shown is for a one-step increase in participation of behavior (e.g. from 1 to 2 days per week to 3–5 days per week)

^b^Higher values indicate higher levels of CMV knowledge

^c^Responses on a 7-point Likert scale where 1 = Strongly Disagree and 7 = Strongly Agree

^d^Behavior-specific perceived behavioral control and response efficacy use for each model

^e^Responses on a 5-point Likert scale where 1 = Not at all effective and 5 = Very effective

### The effect of loss frame messaging among women who perceived they were at risk

It was hypothesized that among those who see themselves at risk for CMV infection, the loss frame would be associated with increased behavioral intention (Hypothesis 3). Among the subset of women who agreed or strongly agreed that they were at risk for CMV infection (*n* = 471), there was no association between framing and overall behavioral intention (*p* = .20) in the unadjusted model or the adjusted model (*p* = 0.70). However, among this same group of women, the mean susceptibility score was higher for those in the most common frame (*M* = 5.01; *SD* = 0.83) compared to the small chance frame (*M* = 4.82; *SD* = 0.89; *p = 0.02*). Similarly, the most common frame was also associated with higher levels of perceived risk with a mean level of 2.74 (*SD* = 1.01) compared to 2.50 (*SD* = 1.17) in the small chance frame (*p* = 0.02). There were no differences in mean risk level between overall loss frames (*M* = 2.64; *SD* = 1.15) and gain frames (*M* = 2.60; *SD* = 1.05).

## Discussion

This study was undertaken to explore the effectiveness of message framing in communicating with women of child-bearing age about how to reduce CMV infection. There were no differences among sample demographics for the four message frames, therefore any difference between the groups with regard to behavioral intention or other constructs should have been due to the messaging. Results showed that message framing had no direct effect on overall behavioral intention. However, there were main effects for knowledge, message credibility, perceived severity, response efficacy and perceived behavioral control. Additionally, interactions with gain and loss frame were noted for behavioral control and response efficacy with associated increases in behavioral intention.

Consistent with other CMV research, we observed high rates of participation in CMV risk behaviors, particularly for sharing food, utensils and cups with children and kissing a child on the lips [15, 16]. Although these behaviors were common, behavioral intention was generally high, indicating that women were willing to modify these behaviors to protect their unborn child. It may be that because CMV awareness was relatively low, exposure to any information regardless of whether it was presented as gains or losses resulted in increased behavioral intention.

The lack of between-group differences for behavioral intention is similar to the results in a meta-analysis on prevention behavior-related messages which observed no difference between gain and loss framing in persuasiveness [37]. Likewise, other studies have found no effect with gain and loss messages regarding vaccination behaviors and intentions to perform behaviors [38, 39]. O’Keefe and colleagues suggested that gain and loss framed messages may not be effective for prevention-related behaviors because outcomes are not certain [37]. This may be true for CMV messaging as well. Although studies have shown that the prevention behaviors included in the fact sheet can reduce CMV infection [14], there are no data that indicate the certainty or magnitude of risk reduction that may occur as a result of doing those behaviors. The absence of between-group differences may also be due to the possibility that subtle messaging differences about losses and gains could have been overshadowed by the CMV information which would have been new to the majority of respondents.

Response efficacy, perceived behavioral control, perceived severity of a CMV infection, message credibility and CMV knowledge were all associated with increases in behavioral intention, though at varying levels. However, it appears that a woman’s perception of effectiveness of the behavior to reduce risk is most influential in her behavioral intention. In other research, a woman’s perception about the effectiveness of the flu vaccine influenced whether or not she received the vaccine [38]. In our study, the perception of effectiveness varied across behaviors with respondents feeling that hand hygiene was most effective at reducing risk of CMV infection. Not sharing food or utensils were seen as less effective than handwashing. Half of the respondents felt that not kissing a child on the lips was very effective (data not shown). Interestingly, the fact sheet did not mention the effectiveness of performing the CMV prevention behaviors, suggesting that people have predetermined beliefs about these behaviors that influence perceptions of efficacy. This may indicate that response efficacy is a function of other constructs that if identified may be modifiable.

The observed interactions that varied between gain frame and perceived behavioral control, and loss frame and response efficacy, may indicate that framing has the potential to differentially impact some subsets of women. A study about sun protection behaviors also observed an interaction between framing and response efficacy for one of three sun protection behaviors [31]. However, loss frame was more persuasive among those with low response efficacy and gain frame was more persuasive for those with high response efficacy [31]. This differs from our finding that behavioral intention increased with increasing levels of response efficacy, but the increase was more pronounced for those in the loss frame. It is unclear whether the differences between these two studies reflect differences in populations or the behaviors studied, or some other factor that differs between CMV prevention and sun protection behaviors.

Although behavioral intention scores were in the positive direction, intention was not equal across all behaviors. A smaller percentage of women reported intention to change sharing behaviors or kissing a child on the lips compared with washing hands. Possible explanations for the relative unwillingness to modify these behaviors (sharing and kissing a child on the lips) may be because these require the cooperation of others and are rooted in cultural norms which could have significant mental and emotional costs if changed. For example, parents often share cups, utensils and food with their child for convenience or to model appropriate eating practices [40]. Kissing a child on the lips is a very common and valued expression of affection [41]. Yet, these are two high-risk behaviors because of the potential sharing of saliva that have high CMV viral loads [3]. Though potentially difficult to change, there have been shifts in cultural norms related to other maternal-child health behaviors such as putting a child to sleep on his/her back [42] and using car safety seats [43].

Additional research could explore how framing interacts with predictors of behavioral intention in various populations and across various types of CMV prevention behaviors. Nevertheless, it is important to recognize that regardless of frame, increased response efficacy, behavioral control, and perceptions of severity were associated with increases in behavioral intention. This suggests that although the complex interaction between framing and these constructs is not fully understood, messaging focused on these constructs may influence behavioral intention. Additionally, recent research on message framing suggests that personal motivating factors such as preferences for self-regulation (e.g., pleasure vs. pain or rewards vs. punishments) may moderate message framing effects [44–46]. This is an unexplored area in CMV research.

Further, the differential impact of messaging on actual behavior change is not known. Additional areas of inquiry should include the testing of CMV messaging strategies in a sample of women who are aware of CMV in order to determine if any information about CMV versus tailored CMV messages influence intention and change. Furthermore, longitudinal research to determine the relationships between behavioral intention and realized behavioral change would be useful.

### Limitations

The study measured only perceived behavioral intention to follow guidelines and not actual behavior. There is no indication that intention to do CMV prevention behaviors will lead to behavior change. However, numerous research studies have demonstrated that intentions are good predictors of actual behavior [47, 48]. The data are self-reported and women may have over- or understated what they do, though the rates of behavior are similar to other CMV studies [15, 16]. Intention to change behavior was measured immediately after seeing the fact sheet and may have suffered from a ceiling effect. There was no control group with which to make comparisons.

Our demographic distribution was similar to other national survey panels studying CMV [15]. The study sample was primarily white with at least some college education. The percent of women with a college degree or greater is higher than the US estimate of 30% [49]. The median US household income is $53,000 (US) [50] and though we cannot directly compare with this study’s income categories, the distribution indicates that our sample maybe be more wealthy. CMV seroprevalence rates are highest among groups with lower socioeconomic status and certain racial-ethnic minority groups [51]. CMV awareness also varies by demographic variables [15]. This data may not be reflective of cultural norms and practices of all racial and ethnic groups or may not represent groups that could benefit the most from CMV messaging. It is possible that these panel women may not represent all women who are pregnant or thinking about becoming pregnant.

## Conclusion

Congenital CMV infection is common and can lead to negative health outcomes. Because awareness is low, and severity is high, determining how to construct CMV prevention messages for optimal influence is of utmost importance. Framing messages by what women have to gain or lose by participating in CMV prevention behaviors does not make a difference in overall behavioral intention. In this study, perceived behavioral control and response efficacy were most predictive of behavioral intention. Additionally, framing interacts with perceived effectiveness of CMV prevention behaviors and individual perceptions of behavioral control to influence intention. There may be other factors that are more persuasive when trying to influence women’s behaviors that may reduce the sharing of a young child’s saliva which has high CMV viral loads. Future messaging that focuses on increasing perceived behavioral control and response efficacy, particularly for the kissing and sharing behaviors, may result in greater gains in intention.

## Abbreviation

CMV: Cytomegalovirus
